# The impact of a non-functioning pituitary adenoma on life – A qualitative study of patients’ experiences

**DOI:** 10.1371/journal.pone.0345909

**Published:** 2026-03-31

**Authors:** Nasrin Al-Shamkhi, Britt Edén Engström, Eva Rask, Agneta Anderzén-Carlsson

**Affiliations:** 1 Endocrinology and Mineral Metabolism, Department of Medical Sciences, Uppsala University, Uppsala, Sweden; 2 School of Medical Sciences, Faculty of Medicine and Health, Örebro University, Örebro, Sweden; 3 University Health Care Research Centre, Faculty of Medicine and Health, Örebro University, Örebro, Sweden; Fondazione Policlinico Universitario Agostino Gemelli IRCCS, ITALY

## Abstract

**Purpose:**

Most studies regarding quality of life in patients with non-functioning pituitary adenoma are based on general questionnaires that might not capture disease-specific aspects, making further exploration of patients’ experiences with non-functioning pituitary adenoma necessary. This study aimed to describe how patients that have undergone surgery due to non-functioning pituitary adenoma experience the effects of the disease on their life.

**Methods:**

Semi-structured interviews were held with eight participants who had undergone surgery due to non-functioning pituitary adenoma. Participants were recruited from an outpatient endocrinology clinic at a tertiary hospital in Sweden. Inductive qualitative content analysis was used.

**Results:**

The analysis identified an overarching theme, *Life has changed but not necessarily for the worse*, and three subthemes: *The knowledge about the tumour evoked existential concerns*, *Suffering became a part of life* and *Finding comfort in a new everyday life.*

**Conclusion:**

The findings show that the participants’ lives have changed due to non-functioning pituitary adenoma. While this change was not always for the worse, the quality of life was negatively impacted for some, despite optimal treatment. A changed view on life and general trust in the healthcare system could temper the impact of non-functioning pituitary adenoma. Moreover, communication between healthcare professionals and patients remains a central aspect of patients’ experiences.

## Introduction

Non-functioning pituitary adenomas (NFPAs) are one of the most common pituitary lesions, with a standardised incidence rate of 1.8 in 100 000 [1]; moreover, increased prevalence and standardised incidence have been reported [2]. A proposed terminology change regarding pituitary adenoma has rendered a debate [3]; hence, in the latest World Health Organization classification the name nonfunctioning pituitary neuroendocrine tumour is used in parallel with NFPA [4].

While NFPAs are benign neoplasms, they can compress the optic chiasm due to the pituitary’s location, giving rise to visual disturbances, which is an indication for treatment. The first-line treatment is transsphenoidal surgery, which may resolve visual disturbances caused by NFPA. Surgical complications, such as cerebrospinal fluid leak or meningitis, are unusual [5]. After transsphenoidal surgery, rhinologic symptoms can be present for at least 6 months [6], along with anosmia [7]. A substantial proportion of patients, up to 80%, have postoperative tumour remnants [8,9]. In case of a significant or growing tumour remnant, radiotherapy or a re-operation might be required [8].

The symptoms of NFPA can be diffuse and non-disease-specific, which may cause delays in diagnosis [10]. In addition to visual disturbances, NFPA can affect the hormonal pituitary function, causing hormonal deficiencies and associated symptoms that require lifelong hormone-replacement therapy. Preoperative hormonal deficiencies may resolve after surgery; however, new deficiencies can occur postoperatively or after radiotherapy [11]. Hence, a NFPA diagnosis implies a state of chronicity, even if the NFPA is surgically removed. In the Swedish context, patients usually have yearly follow-ups to evaluate their hormone status, tumour remnants or regrowth, and any ongoing hormone-replacement therapy.

Most data regarding health-related quality of life (HR-QoL) in patients with NFPA are based on various non-disease-specific questionnaires, and the results are diverse [12–14]. In a systemic review, mainly based on questionnaire data, some studies reported that quality of life was decreased in patients with a NFPA compared with healthy controls and reference values, while others did not [13]. Although qualitative studies on this topic are scarce, it has been noted that the negative impact of a NFPA on quality of life remains postoperatively. One study reported a changed personality and found that other aspects of daily life were also affected [15]. For instance, even though NFPAs are benign in more than 99% of cases, the progression of a tumour remnant or tumour recurrence has been described as a source of concern and fear [15,16]. In a focus group study including patients with NFPA, participants reported several physical, psychological and social issues that are not addressed by general non-disease-specific questionnaires [16]. This finding indicates that HR-QoL in patients with NFPA is not fully unravelled by general questionnaires, yet there is still no validated disease-specific questionnaire that is widely implemented. The need for more insight in this area was recently recognised when patients and healthcare professionals chose the impact of pituitary surgery on quality of life as a research priority [17].

To further understand how the diagnosis and treatment of a NFPA affects patients’ quality of life, there is a need for qualitative studies exploring patients’ experiences, which was the aim of the present study.

## Materials and methods

### Participants

To include the intended population, participants were recruited from an endocrinology and diabetes outpatient clinic at a Swedish tertiary hospital. Patients 18 years or older who had undergone surgery due to NFPA between January 1, 2011, and December 31, 2018, were eligible for the study. The timeframe was set to ensure that the participants had reached a stable phase of the pituitary disease and had received healthcare in accordance with current routines. According to the clinical routines at that time point, surgery was performed at another university hospital approximately 170 kilometres from the patients’ tertiary hospital. If the postoperative course was uncomplicated, the patient was transferred back to the tertiary hospital on postoperative day 3–5 to be admitted for a couple of days at the tertiary hospital.

The core principal of the recruitment was to employ a heterogenous sample regarding age and gender in a well-targeted group of patients. It was recognised that to achieve heterogeneity and thereby contribute to the credibility of the study, patients born in different decades had to be purposely chosen from the outpatient clinic’s waiting list. Thus, in practical terms, if a decade had been repeatedly represented amongst enrolled participants, the next patient on the list born in a different decade was chosen. A nurse at the outpatient clinic sent written study information and consent forms to eligible patients. If there was no response after two weeks, the nurse called the patients to remind them about the study and respond to any questions. To enable broad inclusion, the participants could be provided with an interpreter if needed.

Eight participants were included: four men and four women (Table 1). No medical data on the participants was retrieved from the medical charts, instead it was gathered during the interviews. Included study participants represent patients that have undergone surgery due to NFPA, mostly reporting a transsphenoidal route, some describing pituitary deficiency preoperatively and/or postoperatively including substituted growth hormone deficiency. Amongst the included participants there are cases of reoperation and/or radiotherapy treatment due to significant postoperative tumour remnant.

**Table 1:** Characteristics of study participants.

Gender	N
*Male*	4
*Female*	4
**Age**, years median (min-max)	70.5 (39-83)
**Postoperative pituitary failure**	7
**Marital status**	
*Single*	2
*Married*	5
*Living apart*	1
**Highest degree of formal education**	
*Elementary school*	2
*Upper secondary school*	3
*University*	1
*Unknown*	2

The sample size was repeatedly evaluated during the study. The study applied an exploratory approach and did not use a theory which usually implies a need of more participants. We found that the quality of the interviews was strong; the interviews were purposeful and showed a sufficient variation. New insights emerged from the interviews and during the last interviews, data regarding the aim of the study began to reoccur. Considering the quality of the dialogue, the aim and the explorative design of the study, it was determined that information power [18] had been reached, and recruitment was stopped.

### Interviews

The recruitment period started on the 3rd of December 2021 and ended the 20th of September 2022. All participants signed a written informed consent form prior to their interview. The participants were interviewed individually. One interview was held via a digital secure platform provided by the hospital, while the others were held at the endocrinology and diabetes outpatient clinic. The interviews were semi-structured. An interview guide was used, with questions on how the participants perceived the pituitary disease, their quality of life and whether it had changed since they received the diagnosis, and their perception of the healthcare they had received. There was also a section with questions regarding demographic data including medical data such as relapsed time since surgery, pituitary deficiency and hormone replacement-therapy. All interviews were conducted in Swedish and digitally recorded; no participants required an interpreter. At the time of the interviews, the interviewer (NAS) was doing a residency at the outpatient clinic but had no ongoing professional relationship with the participants. The median interview length was 56 minutes (range: 47–64 minutes). One to two weeks after each interview, NAS called the participants to inquire about any questions or comments the interview might have evoked. None of the participants raised any new issues after the interviews. The interviewer asked one participant to clarify an answer given during the interview.

### Qualitative content analysis

Inductive qualitative content analysis was used to analyse the data [19,20]. The aim of the analysis was to identify both manifest and latent content of the data, to reach a deeper understanding of the participants’ experiences of life and functioning after surgery for NFPA. After the interviews had been transcribed verbatim, NAS read the interviews several times to become familiarised with the text. Every interview was identified as a unit of analysis [19]. Through dialogue between NAS and the co-author, AAC, the transcribed interviews were condensed to meaning units and subsequently manifest codes. The authors avoided too long or short meaning units, since these can include multiple meanings or contribute to a segmentation, respectively [19]. In a separate step, identical or close-to-identical codes were merged. Moving forwards, homogeneous codes were clustered together, based on their manifest content, to create different non-overlapping categories that were internally homogeneous. In dialogue between NAS and AAC, who is a registered nurse experienced in qualitative methods but with no prior experience of the patient group, the data was evaluated for the latent content. To illustrate the latent content three subthemes and an overarching theme were identified. Two senior specialists in endocrinology (ER and BEE) from two different university hospitals with experience in pituitary diseases, participated in the evaluation of the identified themes. Since qualitative interview studies rely on a dynamic process were new insights emerge during data gathering and analysis,which can compromise the study’s dependability, the authors regularly discussed the process as a measure to increase process alignment [19] and NAS repeatedly went back to data to ensure that the codes and categories remained true to the narratives. The credibility was strengthened by cautious selection of the size of meaning units, but also incorporating quotations to reflect the participants’ voices [21] and illustrating the analysis process in Table 2, the latter also contributing to the dependability [19]. No member checking was performed. The qualitative data analysis software NVivo was used for the analysis.

**Table 2:** An example from the analysis process, moving from interview data to latent subthemes.

Interview data	Code	Category	Subtheme
Those around me got a bit..they got scared. My children got very scared ehh.	Relatives worried	It also affected my family and friends	The knowledge about the tumour evoked existential concerns
So that is..I have become a less social person.	Less social after the diagnosis	My everyday life was changed	Suffering became a part of life
It-it was probably the worst, I think, the fact that you have to lie flat..I probably cheated a bit, I think..we put my head up a bit.	The worst to lie down still and flat due to cerebrospinal fluid leak.	The treatment was a struggle for me	Suffering became a part of life
**Interviewee:** Maybe you do not fancy that, maybe you find other things more important. **Interviewer:** Do you remember if you found other things more important? **Interviewee:** ..Yes, that would be family, relatives and friends and such that is the most important	Maybe other things will be perceived as more important postoperatively	My outlook on life has changed	Finding comfort in a new everyday life
I can feel that I have been through very much with this and..yes..it has taught me a lot about life, so I..yes..I am probably more humble	More humble now	My outlook on life has changed	Finding comfort in a new everyday life

### Ethics

The study protocol was reviewed and approved by the Swedish Ethical Review Authority (No. 2021–04228), and the study was conducted in accordance with the Helsinki Declaration [22].

One potential consequence of participating in the study was that the interviews could have evoked unpleasant feelings or memories. If required, participants would have been offered a referral to a counsellor.

Even though we did not enrol patients that the interviewer, who then was a resident at the outpatient clinic, had any ongoing professional relation to there is an inherent power imbalance in doctor-patient interactions that can affect the dynamics in the interviews. Participants might have felt reluctant to address negative aspects of the healthcare or on the contrary, felt pressured to participate and share more information then they wanted to. Besides not enrolling patients that NAS recognised an ongoing professional relation with, NAS did not wear clinical attire during the interviews and did not discuss medical issues in detail during the interviews.

## Results

### Life has changed but not necessarily for the worse

The pituitary disease brought change to the participants’ lives. To varying degrees, existential concerns were evoked, and the patients suffered due to the pituitary disease and its treatment. Still, the change was not necessarily for the worse. The participants commented that they had also found comfort in the changed life they were now living (Table 3).

**Table 3:** Themes, subthemes and categories identified in the analysis.

Theme	Life has changed but not necessarily for the worse		
**Subthemes**	*The knowledge about the tumour evoked existential concerns*	*Suffering became a part of life*	*Finding comfort in a new everyday life*
**Categories**	-It came as a bit of a shock to me	-The treatment was a struggle for me	-Today I feel safe
	-It also affected my family and friends	-My everyday life was changed	-My outlook on life has changed
	-It is still in my head		

### The knowledge about the tumour evoked existential concerns

The diagnosis itself and, later, the knowledge about a postoperative tumour remnant evoked concern in some cases. The participants were not the only ones affected by the diagnosis, as their friends and family were also concerned; this further troubled the participants. Yet the participants found support in friends and family and described having faith in their doctors.

#### 3.2.1. It came as a bit of a shock to me.

For some participants, the diagnosis was a shock. One participant described crying and screaming upon learning what the initial computerised tomography scan had revealed. The participant had not been informed about the benign nature of the tumour and thus awaited the magnetic resonance tomography with fear.

While some participants reported that the most important thing had been to get rid of the tumour, others stated that the diagnosis had brought various concerns regarding the surgical outcome and potential surgical complications, which could affect their life. For instance, one participant revealed thoughts about how the children would react if the participant did not survive. Others feared losing their vision due to the NFPA or the surgery itself, so they chose to go on long-awaited travels while their vision remained intact.

One knew- one could not know what would happen afterwards [the surgery], like.. in what condition one would be. (Interview 4)

At the time of the interviews, some participants could no longer recall what questions they had asked the neurosurgeon, even though they remembered that the diagnosis had brought on troubling thoughts and questions. However, some reported that they continued to ponder aspects of the pituitary disease that still affected them in daily life. Those who had only had subtle symptoms prior to the diagnosis and had not worried about their health or thought about being ill found themselves engaged in new thoughts concerning their health after receiving the diagnosis.

Certainly, I have thought much more after finding out that I have it [the tumour]… (Interview 6)

#### 3.2.2. It also affected my family and friends.

The diagnosis affected not only the participants but also their family and friends. The participants reported that their family and friends were worried, and seeing their relatives upset or worried was troublesome for the participants.

She [grandchild] was so scared that I was going to die…It is tough to talk about it. (Interview 6)

Participants said that they initially had to calm down their family, and some still did so today. Sometimes the participants themselves had explained the diagnosis and surgical procedure to their relatives. Although this could be demanding, as they sometimes had to adapt the information to fit the receiver, some said it had felt good to share information about their diagnosis instead of carrying the burden alone. Furthermore, relatives and friends were often described as supportive.

#### 3.2.3. It is still in my head.

While the diagnosis initially caused existential concerns, later consequences of a tumour remnant or possible tumour remnant growth were also reported as causes for concern. For example, participants that had been informed about a tumour remnant might be extra vigilant for symptoms related to the tumour or possible tumour growth. Participants also expressed a desire that their tumour remnant would not grow.

Depending on the postoperative information received from the doctor, some participants were baffled upon learning about a postoperative tumour remnant.

…baffling..he [the surgeon] said yes, he was pretty sure that he had removed all of it [the tumour], he had looked very closely. (Interview 4)

Others were mentally prepared for a possible re-operation, while one participant described only recently thinking about the risk of tumour regrowth, which was not a pleasant thought.

Although patients with tumour remnants had concerning thoughts regarding the remnant and its consequences, the participants generally said that they trusted the medical decisions that had been made. They realised that there was a fine line between tumour removal and life-long complications.

It could cause more damage…if you attempt to remove it instead of leaving it there. So now they keep track of it instead. And if he [the doctor] says so, yes, then I am calm. (Interview 5)

### Suffering became a part of life

The participants described how the pituitary disease had brought varying degrees of suffering into their lives. Different aspects of the treatment were a struggle, such as awaiting surgery or the results from examinations, being away from home, postoperative complications and sometimes feeling uninformed or unheard. The participants also associated the pituitary disease with symptoms that had forced them to change their lives and, in some cases, restrained them from doing what they wished to do.

#### 3.3.1. The treatment was a struggle for me.

For some, their struggle started in the immediate postoperative phase, when it could be painful to remove nasal tamponades and difficult to endure bedrest due to a cerebrospinal fluid leak. The discomfort of postoperative complications had one participant initially regretting undergoing surgery.

So first I thought…I had changed my mind and said, why did I accept this surgery? (Interview 3)

On the other hand, some did not experience any physical struggle during the immediate perioperative period. However, since all surgery and some eventual radiotherapy were carried out at a different hospital a long way from their home, the participants longed for their family and home during their treatment. In addition to finding themselves far from home during radiotherapy, there were complications, some of which were long-lasting.

Participants wished that the doctors had been more informative, sometimes feeling uninformed when it came to postoperative complications or having to find answers to medical questions themselves.

…and the things that were not answered here, I found out anyway. (Interview 4)

Additionally, some described situations where they had felt that the nurses did not understand their issues or had even felt unheard by the healthcare professionals; for instance, one doctor insisted on prescribing a medication, even though the patient described experiencing side-effects from it. One participant described the struggle they had undergone as a consequence of a doctor not acting on the results of a pathological examination.

Poor collaboration between different healthcare organisations, such as in the referral between hospitals or clinics, could result in a struggle for the participants. Sometimes transport back to the tertiary hospital was rushed, or no one was awaiting the patient upon arrival. In cases of postoperative sinus symptoms, the participants had to be referred to yet another clinic, since endocrinologists do not assess rhinologic symptoms, and the department of neurosurgery was based in another hospital.

Waiting was another struggle, and one that was recurrent: first, the participants had to wait for a diagnosis, sometimes after years of symptoms, then for surgery and later for follow-up. Waiting for follow-up evoked questions about what their results would be and/or whether they had been lost in the system, which actually happened to some participants during referral between different departments.

#### 3.3.2. My everyday life was changed.

Due to the pituitary disease, participants reported a wide range of symptoms they experienced pre- and/or postoperatively and associated with the tumour, such as headaches, low energy, physical restrictions due to adrenocorticotropic deficiency, burnout, erectile dysfunction, visual disturbances and anosmia. The symptoms more or less affected their daily life and sometimes restrained them from living their life as before. Hence, the pituitary disease – or, at least, its repercussions – could affect quality of life.

It was really tough, I..I did not know were life was heading, when I could not work properly and I..there was a lot of worry..both for me and my wife and our children as well, yes. (Interview 1)

One participant specifically said that he still ruminated ‘why me?’ when experiencing headaches. Others found that their lives had changed because they had become a different person due to their low energy levels, which they associated with the pituitary disease. This caused them to have to settle for less and reorganise their lives, such as by passing on social events, time with family or a job.

…I would like to be more engaged in that [party], because I think it is fun. But it [the lack of energy] takes away many fun things. (Interview 2)

One participant specifically reported that the participant’s partner had to take on more responsibility for household chores and initially had had a difficult time accepting the participant’s transformation. The fact that the participant had changed due to the pituitary disease and that it had had an impact on the entire family made the participant sad, angry and frustrated.

Visual disturbances could be the only preoperative symptom, as well as the only sign that remained of the diagnosis postoperatively. Among those who did not find their sight improved postoperatively, the remaining visual disturbances were sometimes perceived as a limitation when reading or pursuing a hobby. They had also led to the withdrawal of participants’ driver’s licenses, which was described as troublesome, at least initially.

The participants reported a variety of views on hormone-replacement therapy. They reflected on whether their personality had changed due to the medications and described side-effects they associated with the hormone-replacement therapy, such as sweating, swelling or trouble sleeping; still, they accepted these side-effects, since they could not manage without medication. On the other hand, others did not view hormone-replacement therapy as a problem; they had adapted to taking daily, life-long medication and acknowledged that their well-being depended on it.

Because of the disease, some patients had to interact with the employment agency and/or social insurance agency, which could be troublesome. In addition, the lack of postoperative rehabilitation and of collaboration between the healthcare system and social insurance agency left the participants feeling stuck and frustrated.

### Finding comfort in a new everyday life

Even though the diagnosis had evoked existential concerns and brought suffering, the participants reported that their outlook on life had changed and their quality of life was maintained. They expressed a positive perspective on Swedish healthcare, which – in combination with the fact that pituitary tumours are usually benign – made them feel safe.

#### 3.4.1. Today I feel safe.

Receiving a diagnosis after several years of symptoms could be regarded as a relief; some even described it as ‘fantastic’. Knowing what caused their symptoms and experiencing a decrease in symptoms after surgery had contributed to an improved quality of life. Some participants appreciated not having to wait long before being contacted by the endocrinology clinic and not being worried while awaiting their first visit at the clinic.

While the diagnosis evoked a variety of thoughts and concerns, some participants reported that they had not been alarmed or distressed by the diagnosis, since they had been informed early on that pituitary tumours are usually benign. Initially, some focused on the positive aspects of the medical process they were going to embark on, which contributed to not feeling scared. Others described accepting the situation, as they were unable to change it. The participants stated that they had not been worried about the surgical procedure itself. They trusted the experienced neurosurgeon they had met prior to surgery and felt trust in Swedish healthcare as well. However, one participant contemplated that the doctors had been ambivalent regarding whether surgery was the correct choice; later, the participant felt that the doctors were worried about the need for a re-operation.

The later yearly postoperative follow-ups were generally not described as fearful or worrying. Instead, the continuity in check-ups made the participants feel safe.

Actually, I think it makes me feel safe, I think it is good, this, with, with continuous check-ups all the time like this, because…probably they will discover something in time, in case there is something, so… (Interview 7)

Although some wished for the doctors to be more informative, participants also reported that their healthcare providers were available when needed and their questions were answered. They felt they could share their concerns with the responsible doctor; for example, one participant reported sharing thoughts regarding tumour growth not with relatives but with the doctor instead. Overall, most participants said they were satisfied with their encounters with the healthcare system.

#### 3.4.2. My outlook on life has changed.

After the diagnosis, some participants described having a newfound realisation that life could change in an instant. Even though the diagnosis could cause concern, suffering and a changed view on life – for some, even a limited daily life – the participants’ perception was that their quality of life was maintained.

Participants described having a changed outlook on life, in which they valued life more and had different priorities than before diagnosis and surgery. For example, one participant described no longer rushing through things and stressing about them. Instead of doing all the ‘musts’, this participant chose to do whatever felt right in the moment.

The participants also reported that they had become more humble, emotional and cautious after surgery.

…I am probably more…humble, maybe, even though I am a soft person, but yet, yet I feel that anything can happen really fast in life. (Interview 8)

Some were thankful that they had been ‘let off easily’, stating that it could have been worse; they viewed their experience as something they could share with others. Others reported that they had not changed and were mostly able to carry on with their lives as usual both pre- and postoperatively; thus, they did not perceive the pituitary disease as a limitation in daily life. For some, this positive outcome was related to a lack of surgical complications. For example, one participant described being thankful for still being able to read and drive a car, since there was little deterioration in the participant’s vision. Others said that they got used to living with visual disturbances.

## Discussion

In this qualitative study aiming to describe how patients that have undergone surgery due to NFPA experience the effects of the disease on their life, it was recognised that the degree to which participants’ lives had changed due to the NFPA varied, with the change not necessarily being for the worse. This variation might partially explain the diversity in outcomes seen in prior quantitative questionnaire based HR-QoL studies [12–14]. The qualitative methodology used herein made it possible to capture a variety of aspects in and degrees to which NFPA can affect quality of life. Even though the diagnosis had evoked existential concerns and brought on suffering to varying degrees, the participants found comfort in a new daily life. Furthermore, besides capturing the struggles of a centralised healthcare when several specialist are involved; the results highlight how information and communication between healthcare professionals and patients remains a central part of patients’ experience and that there is an information gap that the healthcare can partially bridge.

Although the participants in this study described that the diagnosis came as a shock, they also declared that it could still be regarded as a solace to finally find an explanation for the symptoms – a seeming paradox that has been described before [15]. Awaiting the diagnosis was described as part of the struggle the pituitary disease had inflicted on the participants. Therefore, whether the diagnosis was seen as a relief or not might be explained by how lengthy the period with unclear symptoms had been and to what degree the symptoms had affected the patients’ daily life; this is strengthened by data from a previous study that have shown similar implications [15].

The current results show that some of the initial distress that the participants experienced was put to rest after they spoke with the neurosurgeon. The fact that patients’ trust in the neurosurgeon can have a calming effect has been described in previous studies [23]. Although this might be a normal reaction to preoperative information, our results still offer insight into patients’ needs and what the clinician must attend to when offering preoperative counselling. Furthermore, the participants had diverse reactions when learning about postoperative tumour remnants, depending on what they had initially been told after the surgery. Our findings further emphasises that the clinician can affect the patient’s expectations in different phases of the pituitary disease. Despite being assured of the benign nature of NFPA and mostly trusting the medical decisions being made, the participants found the knowledge of a tumour remnant to be a cause for concern, keeping them alert for symptoms that might be due to tumour remnant growth. As described in a previous study [15], tumour remnants remain a cause of fear.

In the midst of the participants’ own concern and worry, they had to explain their diagnosis to others and handle the worry it evoked among family and friends. Even though the participants themselves had to console others, they still found comfort and support in their family and friends. Along with similar reports, the opposite has also been described in prior literature, with patients having a difficult time finding support in relatives and even underplaying their symptoms [24]. The fact that participants became responsible for informing their family and sometimes felt uninformed themselves is indicative of an information gap that is probably not unusual when it comes to rare diagnoses. We believe that the healthcare can partially bridge this gap, for example by encouraging patients to bring a family member to their appointment, offering a contact nurse or support group if available, or providing more written information about pituitary disease.

To maintain a high medical standard, neurosurgery is centralised to Swedish university hospitals but at the time of the study not all university hospitals offered pituitary surgery. Hence, the participants of this study underwent surgery and spent the first postoperative days at a hospital in another county. This was described as part of the participants’ struggle, since they longed for their family; in addition, moving to a different hospital required collaboration between different healthcare providers, which was not always viewed as sufficient. From a praxis perspective, clinicians might not consider the consequences of being away from home; this finding highlights the patient’s perspective when healthcare is centralised and indicates the need for adequate communication between involved healthcare providers.

We found that the diagnosis can evoke existential concerns, and the pituitary disease can have a remaining impact on daily life postoperatively, it is possible that a surge of initial shock or unmet expectations regarding postoperative recovery leads to a deterioration in psychological health. The postoperative recovery phase for NFPA-related surgery has earlier been described as difficult, even leading to suicidal thoughts [15], which healthcare professionals must be vigilant of and address.

The NFPA brought a changed view on life, and some of this study’s participants recognised that their personality had changed, as similarly described in a focus group study [16], but not only in a negative sense. Bearing in mind that the described changes are not exclusive to NFPA, they still might have contributed to the patients’ perception that their quality of life was maintained. Even though the participants entailed that the healthcare provided did not meet all the patients’ needs regarding postoperative information and collaboration, for example, where the former has been addressed in earlier studies as well [23], they still had a positive perspective on Swedish healthcare. This, as well as, the benign nature of the NFPA might have counteracted the shock of the diagnosis and the impact of the pituitary disease on the participants’ quality of life. In a prior study, based on responses to the 36-item Short Form Survey, it has been reported that yearly follow-ups, compared with follow-ups every second year, are associated with a higher mental composite score, whereas the latter are associated with a worse outcome in terms of emotional and mental health [25]. In adherence with that prior finding, the participants in this study described that yearly follow-ups contributed to them feeling safe. Simultaneously, some described how awaiting results temporarily evoked questions and worry.

### Strengths and limitations

Studies with a qualitative approach are scarce. The disease burden and quality of life in patients with NFPA have primarily been described through non-disease-specific questionnaires which has resulted in diverse findings. The strengths of this study’s methodology is the explicit focus on a well-targeted group and the aim to move beyond clinicians’ perspective of the disease burden in patients with NFPA. As such, this study adds to previous studies in that it allows for a detailed insight into the lived experiences of the participants in a systematic way, which can enable a new understanding of patients who have undergone surgery for NFPA [26].

The variation in the participants’ demographic characteristics provided a rich dataset that captured different experiences and aspects of the pituitary disease, which is a strength of this study. The participants represented the heterogeneous clinical outcomes of NFPA: for example, some had undergone re-operations, some had received radiotherapy, some were on hormone-replacement therapy and some had tumour remnants. That said, a greater diversity in the participants’ ages might have brought insight into other aspects of the disease, such as reproductive issues due to hypogonadism as a result of the pituitary disease.

Despite the strengths of this study, there are some limitations to acknowledge. One possible limitation is that we did not seek ethical approval to access the medical charts of the participants to collect additional data of possible importance for judging transferability. Instead, data on time of surgery, surgical approach, deficient pituitary axes and hormonal replacement-therapy was collected during the interviews, which in hindsight perhaps did not provide enough details.

Secondly, only NFPA patients who had undergone surgery under a certain period, the latter to ensure that disease stability had been reached and that the patients had received care according to the same guidelines, were enrolled. This might restrict transferability to the entire group of NFPA patients. With that said, we wanted to target patients who had undergone surgical intervention and the study also captures quality of life preoperatively and for some participants that period spanned over years.

As discussed in the Ethics section, the fact that NAS was part of the staff at the outpatient clinic might have influenced the interviews. Participants might have felt reluctant to address or even downplayed negative aspects of the healthcare they had received or, the opposite, felt pressured to share information. Taking this into consideration, NAS did not wear clinical attire during interviews and did not discuss medical issues in detail during the interviews. Participants did talk about negative experiences during the interviews, indicating some degree of ability to share their perception of the healthcare services they had received.

Since all participants were enrolled from the same outpatient clinic, an institutional bias regarding their experiences of the healthcare might have been introduced. On the contrary, enrolling from one outpatient clinic also creates a common stepping ground for all participants’ journey through the medical system due to NFPA. Co-author BEE and ER, who have long and varied experience of NFPA, recognised the themes which contributes to the study’s credibility. Overall, the researchers’ varied backgrounds and clinical experiences, were valuable for reflexivity and strengthens the study’s credibility and confirmability. To further increase the study’s credibility and dependability, and to allow the reader to evaluate the trustworthiness, Table 2 offers an example of the analysis process. In hindsight, member checking could have contributed to the study’s credibility, but such participant involvement was not included in the ethical application.

## Conclusion

While this study emphasises that NFPA has an impact on patients’ quality of life, this change is not necessarily for the worse. However, clinicians should be aware that NFPA can still affect patients’ quality of life postoperatively, even when the patient is seemingly cured or optimally treated. Being informed, to decrease any information gap, and listened to are central matters that can affect patients’ experiences. Aspects such as patients’ perception of the healthcare and a changed personality and/or different view on life due to the NFPA might contribute to tempering the disease burden, but further qualitative studies are needed to clarify disease-specific needs.

## References

[1] Tjörnstrand A, Gunnarsson K, Evert M, Holmberg E, Ragnarsson O, Rosén T, et al. The incidence rate of pituitary adenomas in western Sweden for the period 2001-2011. Eur J Endocrinol. 2014;171(4):519–26. doi: 10.1530/EJE-14-0144 25084775

[2] Agustsson TT, Baldvinsdottir T, Jonasson JG, Olafsdottir E, Steinthorsdottir V, Sigurdsson G, et al. The epidemiology of pituitary adenomas in Iceland, 1955-2012: a nationwide population-based study. Eur J Endocrinol. 2015;173(5):655–64. doi: 10.1530/EJE-15-0189 26423473

[3] Ho K, Fleseriu M, Kaiser U, Salvatori R, Brue T, Lopes MB, et al. J Endocr Soc. 2021;5(3):bvaa205. doi: 10.1210/jendso/bvaa205 33604494 PMC7874572

[4] Mete O. Pituitary Tumours: Introduction. Endocrine and Neuroendocrine Tumours. 5 ed. Lyon, France: International Agency for Research on Cancer. 2022. https://tumourclassification.iarc.who.int/chapters/53

[5] Murad MH, Fernández-Balsells MM, Barwise A, Gallegos-Orozco JF, Paul A, Lane MA, et al. Outcomes of surgical treatment for nonfunctioning pituitary adenomas: a systematic review and meta-analysis. Clin Endocrinol (Oxf). 2010;73(6):777–91. doi: 10.1111/j.1365-2265.2010.03875.x 20846296

[6] Hallén T, Olsson DS, Farahmand D, Esposito D, Olofsson A-C, Jakobsson S, et al. Sinonasal Symptoms and Self-Reported Health before and after Endoscopic Pituitary Surgery-A Prospective Study. J Neurol Surg B Skull Base. 2021;83(Suppl 2):e160–8. doi: 10.1055/s-0041-1722929 35832966 PMC9272326

[7] Baudracco I, Ekanayake J, Warner E, Grieve JP, Dorward NL. Olfactory outcomes after transsphenoidal endonasal surgery. Br J Neurosurg. 2020;34(1):35–9. doi: 10.1080/02688697.2019.1680798 31709822

[8] Tampourlou M, Ntali G, Ahmed S, Arlt W, Ayuk J, Byrne JV, et al. Outcome of Nonfunctioning Pituitary Adenomas That Regrow After Primary Treatment: A Study From Two Large UK Centers. J Clin Endocrinol Metab. 2017;102(6):1889–97. doi: 10.1210/jc.2016-4061 28323946

[9] Reddy R, Cudlip S, Byrne JV, Karavitaki N, Wass JAH. Can we ever stop imaging in surgically treated and radiotherapy-naive patients with non-functioning pituitary adenoma?. Eur J Endocrinol. 2011;165(5):739–44. doi: 10.1530/EJE-11-0566 21900406

[10] Drange MR, Fram NR, Herman-Bonert V, Melmed S. Pituitary tumor registry: a novel clinical resource. J Clin Endocrinol Metab. 2000;85(1):168–74. doi: 10.1210/jcem.85.1.6309 10634382

[11] Al-Shamkhi N, Berinder K, Borg H, Burman P, Dahlqvist P, Höybye C, et al. Pituitary function before and after surgery for nonfunctioning pituitary adenomas-data from the Swedish Pituitary Register. Eur J Endocrinol. 2023;189(2):217–24. doi: 10.1093/ejendo/lvad104 37551511

[12] Andela CD, Lobatto DJ, Pereira AM, van Furth WR, Biermasz NR. How non-functioning pituitary adenomas can affect health-related quality of life: a conceptual model and literature review. Pituitary. 2018;21(2):208–16. doi: 10.1007/s11102-017-0860-4 29302835 PMC5849670

[13] Andela CD, Scharloo M, Pereira AM, Kaptein AA, Biermasz NR. Quality of life (QoL) impairments in patients with a pituitary adenoma: a systematic review of QoL studies. Pituitary. 2015;18(5):752–76. doi: 10.1007/s11102-015-0636-7 25605584

[14] Vega-Beyhart A, Enriquez-Estrada VM, Bello-Chavolla OY, Torres-Victoria TR, Martínez-Sánchez FD, López-Navarro JM, et al. Quality of life is significantly impaired in both secretory and non-functioning pituitary adenomas. Clin Endocrinol (Oxf). 2019;90(3):457–67. doi: 10.1111/cen.13915 30548674

[15] Simpson J, Heath J, Wall G. Living with a pituitary tumour: a narrative analysis. Psychol Health. 2014;29(2):162–76. doi: 10.1080/08870446.2013.840784 24070069

[16] Andela CD, Niemeijer ND, Scharloo M, Tiemensma J, Kanagasabapathy S, Pereira AM, et al. Towards a better quality of life (QoL) for patients with pituitary diseases: results from a focus group study exploring QoL. Pituitary. 2015;18(1):86–100. doi: 10.1007/s11102-014-0561-1 24682940

[17] Newall N, Valetopoulou A, Khan DZ, Borg A, Bouloux PMG, Bremner F, et al. Identifying research priorities for pituitary adenoma surgery: an international Delphi consensus statement. Pituitary. 2025;28(2):36. doi: 10.1007/s11102-025-01502-7 40042764 PMC11882698

[18] Malterud K, Siersma VD, Guassora AD. Sample Size in Qualitative Interview Studies: Guided by Information Power. Qual Health Res. 2016;26(13):1753–60. doi: 10.1177/1049732315617444 26613970

[19] Graneheim UH, Lundman B. Qualitative content analysis in nursing research: concepts, procedures and measures to achieve trustworthiness. Nurse Educ Today. 2004;24(2):105–12. doi: 10.1016/j.nedt.2003.10.001 14769454

[20] Lindgren B-M, Lundman B, Graneheim UH. Abstraction and interpretation during the qualitative content analysis process. Int J Nurs Stud. 2020;108:103632. doi: 10.1016/j.ijnurstu.2020.103632 32505813

[21] Eldh AC, Årestedt L, Berterö C. Quotations in Qualitative Studies: Reflections on Constituents, Custom, and Purpose. International Journal of Qualitative Methods. 2020;19. doi: 10.1177/1609406920969268

[22] World Medical Association. World Medical Association Declaration of Helsinki: Ethical Principles for Medical Research Involving Human Participants. The Journal of the American Medical Association. 2025;333(1):71–4. doi: 10.1001/jama.2024.21972 39425955

[23] Edem IJ, Banton B, Bernstein M, Lwu S, Vescan A, Gentilli F, et al. A prospective qualitative study on patients’ perceptions of endoscopic endonasal transsphenoidal surgery. Br J Neurosurg. 2013;27(1):50–5. doi: 10.3109/02688697.2012.711497 22844972

[24] Jakobsson Ung E, Olofsson A-C, Björkman I, Hallén T, Olsson DS, Ragnarsson O, et al. The pre- and postoperative illness trajectory in patients with pituitary tumours. Endocr Connect. 2019;8(7):878–86. doi: 10.1530/EC-19-0202 31176303 PMC6599075

[25] Crouzeix G, Morello R, Thariat J, Morera J, Joubert M, Reznik Y. Quality of Life but not Cognition is Impacted by Radiotherapy in Patients with Non-Functioning Pituitary Adenoma. Horm Metab Res. 2019;51(3):178–85. doi: 10.1055/a-0850-9448 30861564

[26] Richards L, Morse JM. Readme first for a user’s guide to qualitative methods. 2nd ed. Thousand Oaks Calif.: Sage Publications. 2007.

